# *Pinus flexilis* and *Picea engelmannii* share a simple and consistent needle endophyte microbiota with a potential role in nitrogen fixation

**DOI:** 10.3389/fmicb.2014.00333

**Published:** 2014-07-04

**Authors:** Alyssa A. Carrell, Anna C. Frank

**Affiliations:** Life and Environmental Sciences and Sierra Nevada Research Institute, School of Natural Sciences, University of California, MercedMerced, CA, USA

**Keywords:** bacterial endophytes, 16S rRNA, conifers, *Pinus*, *Picea*, nitrogen, *Acetobacteraceae*, subalpine

## Abstract

Conifers predominantly occur on soils or in climates that are suboptimal for plant growth. This is generally attributed to symbioses with mycorrhizal fungi and to conifer adaptations, but recent experiments suggest that aboveground endophytic bacteria in conifers fix nitrogen (N) and affect host shoot tissue growth. Because most bacteria cannot be grown in the laboratory very little is known about conifer–endophyte associations in the wild. *Pinus flexilis* (limber pine) and *Picea engelmannii* (Engelmann spruce) growing in a subalpine, nutrient-limited environment are potential candidates for hosting endophytes with roles in N_2_ fixation and abiotic stress tolerance. We used 16S rRNA pyrosequencing to ask whether these conifers host a core of bacterial species that are consistently associated with conifer individuals and therefore potential mutualists. We found that while overall the endophyte communities clustered according to host species, both conifers were consistently dominated by the same phylotype, which made up 19–53% and 14–39% of the sequences in *P. flexilis* and *P. engelmannii,* respectively. This phylotype is related to *Gluconacetobacter diazotrophicus* and other N_2_ fixing acetic acid bacterial endophytes. The pattern observed for the *P. flexilis* and *P. engelmannii* needle microbiota—a small number of major species that are consistently associated with the host across individuals and species—is unprecedented for an endophyte community, and suggests a specialized beneficial endophyte function. One possibility is endophytic N fixation, which could help explain how conifers can grow in severely nitrogen-limited soil, and why some forest ecosystems accumulate more N than can be accounted for by known nitrogen input pathways.

## INTRODUCTION

Bacterial endophytes inhabit the below- and aboveground tissues of all terrestrial plants examined and can affect plant physiology and growth under normal and stressed conditions. Endophytic bacteria can stimulate plant growth directly through production of phytohormones and volatiles (Arshad and Frankenberger, 1991; Ping and Boland, 2004; Hardoim et al., 2008), enhance nutrient acquisition (Hurek et al., 2002; Boddey et al., 2003), and suppress stress-induced ethylene synthesis (Glick, 2004). Bacterial endophytes have also been found to protect against disease (Conn et al., 2008), and against abiotic stress such as salinity, and heavy metals (Idris et al., 2004; Mayak et al., 2004). At the ecosystem level, bacterial endophytes can persistently alternate soil biogeochemical cycles through the support of invasive plants (Rout et al., 2013).

Research to date on bacterial endophytes has focused mainly on agricultural ecosystems (Hallman et al., 1997), and to some extent, on invasive plants (Rout et al., 2013). Our knowledge of the role, diversity, and transmission of bacterial endophytes colonizing native plants is still limited. However, plant-beneficial endophytic properties are likely to have evolved in, and continually influence plants in natural ecosystems. A better understanding of the bacteria that inhabit wild plants has the potential to impact our understanding not only of basic plant physiology, but also of whole ecosystem processes such as carbon (C) and N cycling.

While it is well established that many forest conifers depend on associations with mycorrhizal- (Smith and Read, 2008) and foliar endophytic fungi (Carroll, 1988; Arnold et al., 2003; Higgins et al., 2011), our understanding of the bacterial endophytes of conifers is limited (Pirttilä and Frank, 2011). Bacteria have been isolated from the interior of roots, stems, needles, seeds, and tissue culture of conifers (Pirttilä et al., 2000; Cankar et al., 2005; Izumi et al., 2008; Bal et al., 2012). Bacteria in the genus *Methylobacterium* likely play a role in the shoot tissue development of *P. flexilis* (Pirttilä et al., 2004, 2005). N_2_ fixing bacteria isolated from stems and needles of *Pinus contorta* have been found to increase the uptake atmospheric N_2_ in inoculated seedlings relative to control seedlings (Bal and Chanway, 2012; Anand and Chanway, 2013). A similar experiment on poplar clones inoculated with a consortium of N_2_ fixing bacteria suggests that N allocated to the leaves and stem in the poplar clones were derived largely from biological nitrogen fixation (Knoth et al., 2013). These results raise numerous questions: do non-nodulated trees in natural ecosystems acquire their N from endophytic N_2_ fixation? If so, are associations random or stable, and do single species or consortia of N_2_ fixing bacteria fix N_2_? Finally, does endophytic N_2_ fixation, along with other recently discovered nitrogen input pathways (DeLuca et al., 2002; Morford et al., 2011; Vitousek et al., 2013) explain the hidden input of N in temperate and boreal forests (Bormann et al., 1993, 2002; Binkley et al., 2000)? Unfortunately, a culture-based and experimental approach to answering these questions may not yet be feasible. Large discrepancies in the composition between cultured and uncultured endophytic communities (Ando et al., 2005) suggest that most bacterial endophytes may resist cultivation. In addition, there is no guarantee that an isolated species represents a dominant member of the endophytic community.

In general, sequence-based methods may be necessary to uncover some of the hidden symbioses in terrestrial ecosystems (Poole et al., 2012). High-throughput sequencing of the 16S rRNA can be used to identify the core subset of bacterial taxa present in the majority of host individuals, habitats, seasons, environments or developmental stages (Qin et al., 2010; Martinson et al., 2011; Lundberg et al., 2012; Rastogi et al., 2012; Sharp et al., 2012). Such core taxa often provide specialized beneficial functions to a host. In insect guts for example, a simple (i.e., consisting of few species) and consistent (i.e., present in all host individuals examined) microbiota typically plays a role in host nutrient acquisition (Engel and Moran, 2013). Once established, the role of core uncultured members of the community can be further evaluated using *in situ* methods (Sharp et al., 2012; Woebken et al., 2012).

Here, we used pyrosequencing of the 16S rRNA gene with the goal of identifying the core subset of bacterial species inside needles that are consistently associated with individuals of a conifer host species. Specifically, we asked whether the needle microbiota is conserved across individuals and stands of the high-elevation, stress-tolerant conifer species *P. flexilis* (limber pine) growing within one geographic area (Niwot Ridge in the Front Range of the Rocky Mountains, CO, USA), and whether the *P. flexilis* needle microbiota is shared with the co-occurring species *P. engelmannii* (Engelmann spruce).

This first comprehensive analysis of the bacterial endophyte microbiota in a gymnosperm revealed that the composition of *P. flexilis* and *P. engelmannii* needle endophyte communities is simple at the species level; that both conifer hosts are consistently dominated by potential N_2_ fixing taxa in the family *Acetobacteraceae*; and that the microbiota is largely shared between the two host species. We discuss the implications of these results in light of recent findings that bacterial endophytes appear to fix N_2_ inside conifer tissues in an experimental system.

## MATERIALS AND METHODS

### SAMPLE COLLECTION AND STERILIZATION

Needles were collected from 14 trees at two elevations in the subalpine forest at Niwot Ridge, CO in September 2009—at tree line (TL) and near the local warm edge (WE) limit of the *P. flexilis* and *P. engelmannii* distributions (**Table 1**). To assess the difference in endophytic communities across locations, six *P. flexilis* and six *P. engelmannii* trees were sampled at two separate WE stands (WE1 and WE2), approximately 0.5 km apart. To contrast inter- and intra tree variation in the endophytic community, one individual of each tree species at each stand was sampled in triplicate. Additionally, two *P. flexilis* and two *P. engelmannii* individuals were sampled at treeline, the upper elevation limit of upright tree growth. From each sampled tree, approximately 10 g of needles were removed with a sterile razor blade, placed in a sterile bag, and shipped to the University of California, Merced at 4°C for sterilization and extraction. Needles were sterilized through submersion in ethanol for one minute, 30% hydrogen peroxide for 3 min, followed by three rinses with sterile deionized water, and stored at –20°C. The final rinse after sterilization was saved to verify sterility (Izumi et al., 2008).

**Table 1:** Samples successfully characterized by 16S rRNA in this study, along with the number of sequences after sequence quality control and removal of plant DNA.

Sampling location	Tree species	Tree ID	Sample	No. of Sequences	No. of 97% phylotypes
WE1	*P. flexilis*	01	WE.01	1736	141
			WE.02	1838	131
			WE.03	1826	138
		02	WE.04	1212	162
		03	WE.05	1176	128
	*P. engelmannii*	04	WE.11	1668	125
		05	WE.12	2098	180
WE2	*P. flexilis*	06	WE.06	1750	114
			WE.07	1804	106
			WE.08	1809	117
		07	WE.09	1602	146
		08	WE.10	875	101
	*P. engelmannii*	09	WE.13	1709	157
			WE.14	1055	134
			WE.15	1254	164
		10	WE.16	1287	150
TL1	*P. flexilis*	11	TL.01	1199	31
		12	TL.02	1332	41
	*P. engelmannii*	13	TL.11	865	58
		14	TL.12	1202	80

### DNA EXTRACTION

Needles were ground to a fine powder in a sterile mortar with liquid nitrogen. In a 2 ml screw cap tube, 800 μl of CTAB solution (1 ml CTAB buffer, 0.04 g of polyvinylpyrollidone, 5 μl of 2-mercaptoethanol) was added to 0.6 g of ground needle material. The tube was then incubated in a dry bath at 60°C for 2 h with intermittent vortexing. After incubation, 0.3 g of 0.11 mm sterile glass beads was added to the tube and the sample was homogenized using a bead beater for 3 min. To remove proteins, an equal amount of chloroform was added to the tube, vortexed, and centrifuged for 10 min at 16 rcf. For precipitation of nucleic acids, the aqueous top phase was placed in a sterile 2 ml snap cap tube with 1/10 volume of cold 3 M sodium acetate and 1/2 volume cold isopropanol and placed in a –20°C freezer for 12 h. The sample was then centrifuged for 3 min at 16 rcf, supernatant decanted, 700 μl of 70% ethanol added, and centrifuged for 10 min. The air-dried pellet was resuspended with 30 μl of DNA resuspension fluid (1.0 M Tris–HCl, 0.1 M EDTA) and stored at –20°C.

### DNA AMPLIFICATION

The extract was used to perform a nested PCR with Golay barcoded, chloroplast-excluding primers, 16S 799f (AACMGGATTAGATACCCKG) and 16S 1492r (TACGGHTACCTTGTTACGACTT; Chelius and Triplett, 2001; Redford et al., 2010), using the thermocycle profile described in Jiao et al. (2006). We used nested PCR to reduce the occurrence of plastid sequences, improve consistency (Hanshew et al., 2013) and minimize non-specific amplification from the barcoded primers that include 454 adapters (Berry et al., 2011). PCR amplification with primer 16S 799f resulted in a mitochondrial product of about 1000 bp and bacterial product of about 750 bp as described in Chelius and Triplett (2001). The bacterial product was then separated and extracted using E-Gel®; SizeSelect^TM^ Gels (Life Technologies, Carlsbad, CA, USA). The extracted bacterial product was amplified with the thermocycle profile described by Jiao et al. (2006) using the barcoded primer set, 799f and 1115r (AGGGTTGCGCTCGTTG), described by Redford et al. (2010) as an optimized primer set for phylogenetic analysis of pyrosequencing reads. The final product was then cleaned, quantified using Nanodrop, and pooled for pyrosequencing. The pooled product was sent to the Environmental Genomics Core Facility at the University of South Carolina for pyrosequencing on a 454 Life Sciences Genome Sequencer FLX machine.

### SEQUENCE ANALYSIS

Sequences were analyzed and processed using the QIIME package (Caporaso et al., 2010b). Briefly, sequences were quality filtered (minimum quality score of 25, minimum length of 200 bp, and no ambiguity in primer sequence) and assigned to their corresponding sample by the barcode sequences. Samples with less than 200 sequences were removed. These included three samples from a *P. engelmannii* WE1 individual, and one sample from one *P. engelmannii* WE2 individual. The remaining sequences were clustered into phylotypes using UCLUST (Edgar, 2010), with a minimum coverage of 99% and a minimum identity of 97%. A representative sequence was chosen for each phylotype by selecting the longest sequence that had the highest number of hits to other sequences of that particular phylotype. Chimeric sequences were detected with ChimeraSlayer and removed before taxonomic analysis (Edgar et al., 2011). Representative sequences were aligned using PyNAST (Caporaso et al., 2010a) against the Greengenes core set (DeSantis et al., 2006). Taxonomic assignments were made using the Ribosomal Database Project (RDP) classifier (Wang et al., 2007). Sequences classified as “Chloroplast” (0.2%) or “Mitochondria” (8%) were removed from the alignment.

**FIGURE 1:**
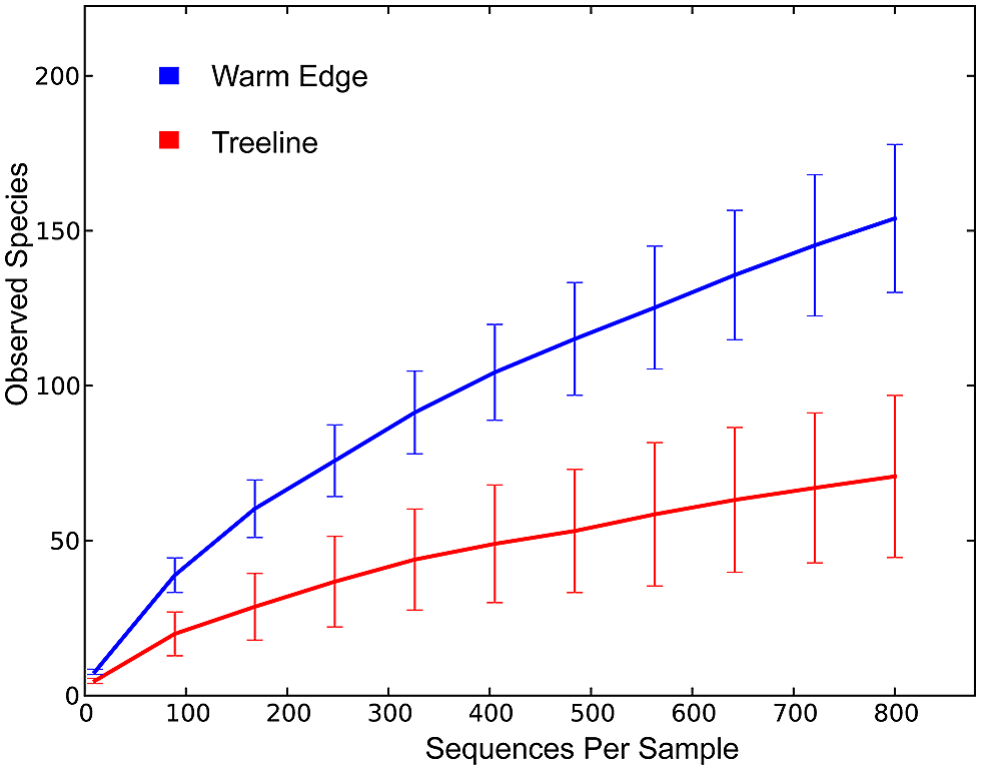
**Rarefaction curves for WE and treeline samples.** There is no apparent asymptote in the rarefaction curves, suggesting that the sequencing depth does not encompass the full extent of phylotype richness in each of the communities. However, the rarefaction curves suggest that the lower number of phylotypes recovered from treeline samples was not due to insufficient sampling. The high and low of the error bars represent one SD away from the mean.

**FIGURE 2:**
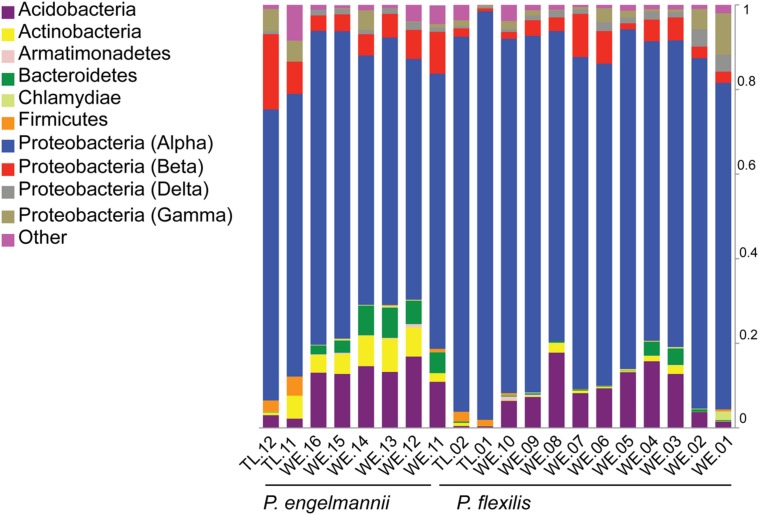
**Relative abundances of various major bacterial phyla and classes recovered from *P. flexilis* and *P. engelmannii* needles.** Relative abundance of phyla (and classes of the Proteobacteria) was calculated as the percentage of sequences belonging to a particular lineage of all 16S rRNA gene sequences recovered from each sample.

**FIGURE 3:**
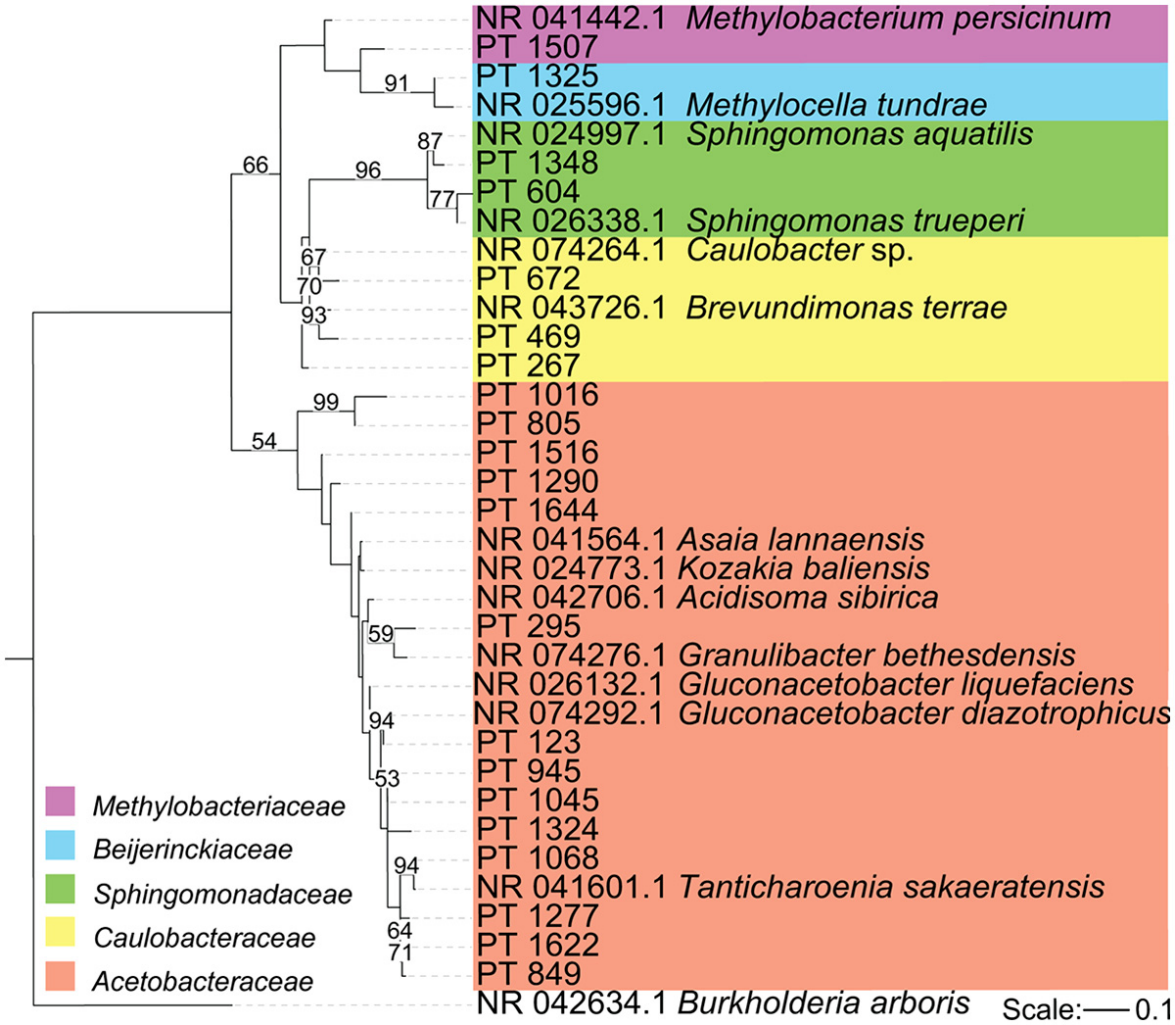
**Phylogeny of major *Alphaproteobacterial* sequences in our samples.** Maximum likelihood phylogeny of *Alphaproteobacterial* sequences that occurred at least 100 times along with the three most closely related sequences from the GenBank 16S rRNA database (accession number indicated). Because our sequences are short (approximately 300 nt), many of our clades have low bootstrap support. Here, only bootstrap values above 50% are displayed. The tree is rooted with *Burkholderia arboris*.

**FIGURE 4:**
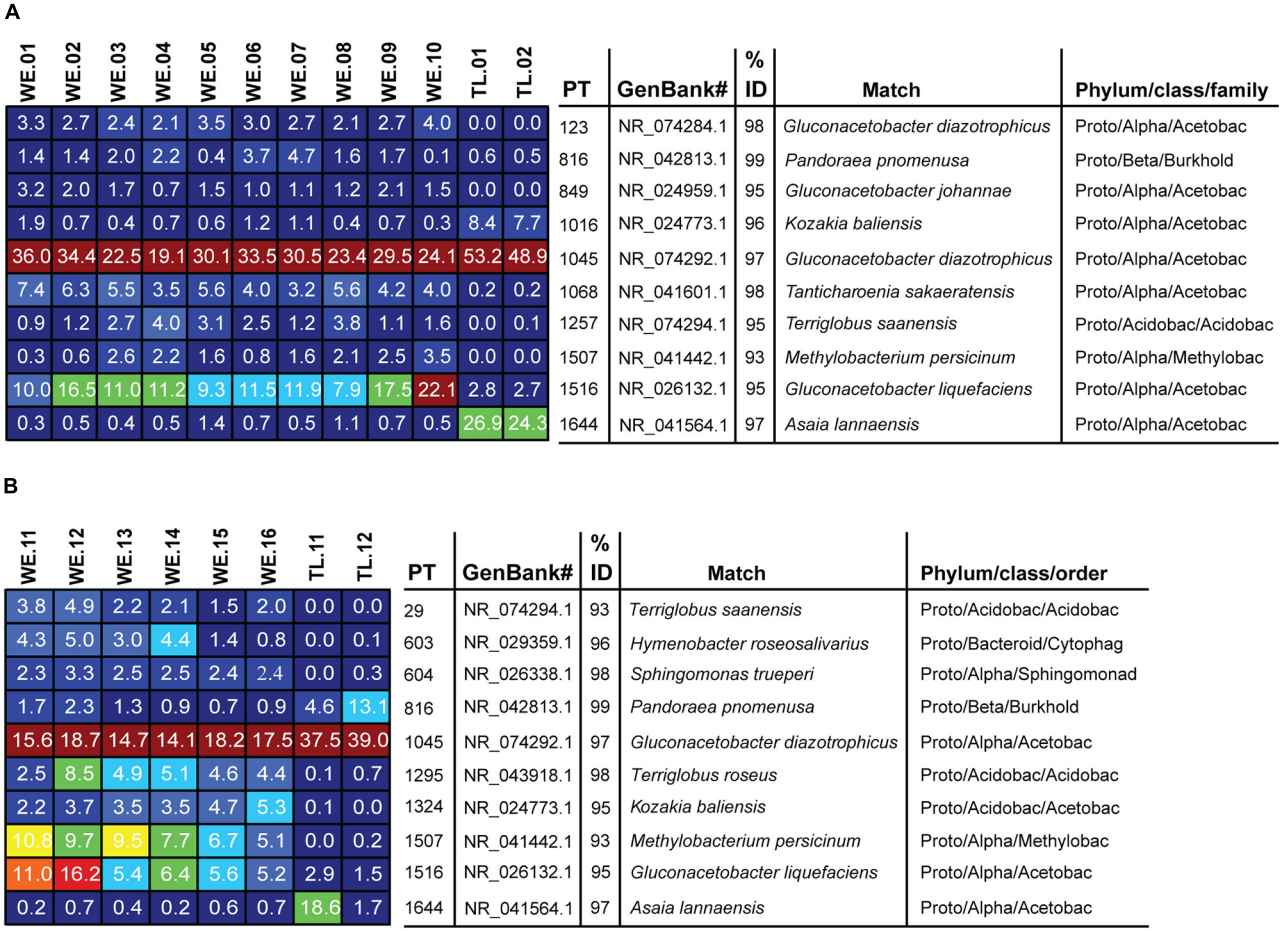
**Heatmap showing the 10 most dominant phylotypes and their average relative abundances as percentages of all sample 16S rRNA gene sequences recovered in our conifer needles samples. (A)** *P. flexilis*, **(B)** *P. engelmannii*. Color tones range from cool (blue) to warm (red) to indicate the lowest to highest relative abundance values. Phylotypes were considered dominant if they were both highly abundant and occurred frequently in samples of a given conifer species. Abbreviations: PT = Phylotype, Acetobac = *Acetobacteraceae*, Acidobac = Acidobacteria/*Acidobacteriaceae*, Burkhold = *Burkholderiaceae*, Bacteriod = Bacteriodetes, Flexibacteraceae, Methylobac = *Methylobacteriaceae*, Cytophag = *Cytophagacae*, Sphingomonad = *Sphingomonadaceae*.

**FIGURE 5:**
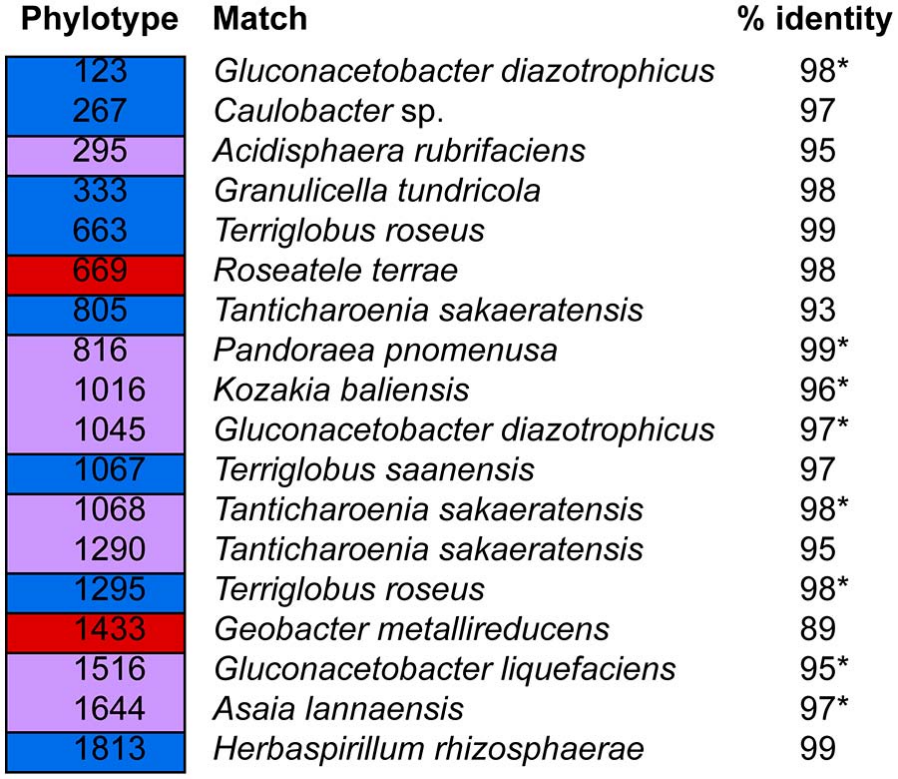
**Shared and host species-specific phylotypes.** Blue: phylotypes found in all *P. engelmannii* samples but not in any of the *P. flexilis* samples. Red: phylotypes found in all *P. flexilis* samples but not in any of the *P. engelmannii* samples. Purple: indicates phylotypes recovered from all samples (i.e., both species). An asterisk indicates that the phylotype is included in **Figure 2**.

**FIGURE 6:**
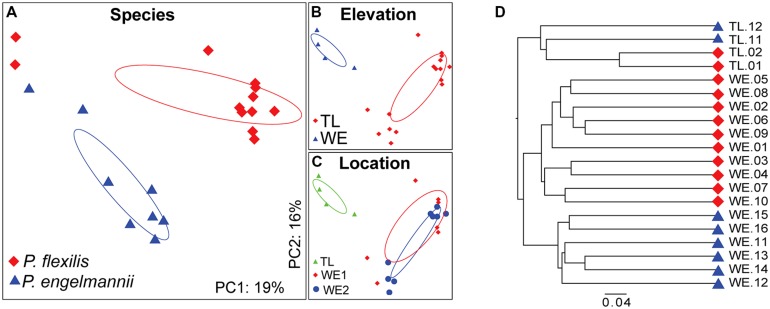
**PCoA and UniFrac analysis of the bacterial communities associated with conifer needles. (A–C)** PCoA of the unweighted UniFrac distance matrix. Points that are closer together on the ordination have communities that are more similar. Each point corresponds to a sample, and shapes correspond to **(A)** host species, **(B)** elevation, and **(C)** location. **(D)** Hierarchical clustering of composite communities of the conifer species. Leaves are labeled by color according to host species: red, *P. flexilis*; blue, *P. engelmannii*.

To compare diversity levels between WE and treeline samples and control for differences in sequencing depth between samples from the two environments, we conducted rarefaction analyses with 800 randomly selected sequences per sample. The rarefaction curves are displayed in **Figure 1**. The relative abundance of bacterial classes in each sample, displayed in **Figure 2**, was calculated as the percentage of sequences belonging to a particular phylum of all 16S rRNA gene sequences recovered from each sample, with the Proteobacteria split into classes. The Alphaproteobacterial phylogenetic tree displayed in **Figure 3** was created by first searching Alphaproteobacterial sequences that occurred at least 100 times in our data against the GenBank 16S rRNA database, using BLAST. The top hit for each sequence was then downloaded aligned from RDP. Our sequences, along with an outgroup sequence (*Burkholderia arboris*) were added to the alignment using ClustalW, before a maximum likelihood tree was inferred using RAxML (1000 bootstrap replicates; Stamatakis et al., 2005). To create the heatmap displayed in **Figure 4**, the heatmap function in QIIME was used. The function visualizes the operational taxonomic unit (OTU) table generated by QIIME (this table tabulates the number of times an OTU is found in each sample). In **Figure 4**, only the 10 most common OTUs (phylotypes) were included. To create **Figure 5**, a heatmap of all OTUs was generated to identify phylotypes unique to each species and shared across all samples, respectively. For each phylotype in **Figures 4** and **5**, the similarity to known isolates was determined though a BLAST search against the NCBI 16S rRNA database. To create **Figure 6**, an approximately maximum-likelihood tree was constructed from the alignment using FastTree (Price et al., 2009). An unweighted UniFrac distance matrix was constructed from the phylogenetic tree. The unweighted Unifraq distances were visualized using principal coordinate analysis (PCoA) and an UPGMA tree was created from the UniFraq distance matrix. Confidence ellipses (95%) were drawn around groups on the PCoA plots using the ordiellipse function of the Vegan package in R (Oksanen et al., 2008).

## RESULTS

### PHYLOTYPES RECOVERED FROM SAMPLES

A total of 388 distinct bacterial phylotypes distributed across 166 bacterial phyla were recovered from 29,297 quality sequences from the 20 samples. These sequence data have been submitted to the GenBank databases under project accession No. SRP033097. On average, each sample yielded 1465 sequences after plant DNA was removed. The number of sequences recovered did not differ greatly between individuals, species, or locations (**Table 1**). The average number of phylotypes recovered also did not vary much within individuals or across species, but varied across locations. An average of 137 endophytic phylotypes were recovered from WE samples while an average of 53 phylotypes were recovered from treeline samples (**Table 1**). Rarefaction curves suggested that the lower number of phylotypes recovered from treeline samples was not due to insufficient sampling (**Figure 1**).

### DOMINANT BACTERIAL TAXA ASSOCIATED WITH *P. flexilis* AND *P. engelmannii* NEEDLES

We found a low degree of intra-individual, inter-individual, and interspecies variability in the taxonomic structure of the endophytic communities of our two conifer species. The majority of detected taxa belonged to two bacterial phyla: Acidobacteria and Proteobacteria (**Figure 2**). The proportion of Acidobacteria varied among samples and accounted for between 2 and 17% in the WE samples, but only 0.5–3% in the treeline samples. All of our samples were dominated by taxa in the Alphaproteobacteria (**Figure 2**). The Alphaproteobacterial phylotypes recovered here belong to five families: *Acetobacteraceae*, *Beijerinckiaceae*, *Caulobacteraceae*, *Methylobacteriaceae*, and *Sphingomonadaceae*.

Among the Alphaproteobacteria, and over all, the *Acetobacteraceae* (acetic acid bacteria) was the most common family recovered in our samples. Eighty-eight out of the total 188 Alphaproteobacterial phylotypes detected belonged to this family, which dominated all samples, accounting for at least 60% of phylotypes detected in each sample. This family is currently classified into 13 genera; *Acetobacter*, *Gluconobacter*, *Gluconacetobacter*, *Acidomonas*, *Asaia*, *Kozakia* (Kersters et al., 2006), *Swaminathania* (Loganathan and Nair, 2004), *Saccharibacter* (Jojima et al., 2004), *Neoasaia* (Yukphan et al., 2005), *Granulibacter* (Greenberg et al., 2006), *Commensalibacter* (Roh et al., 2008), *Tanticharoenia* (Yukphan et al., 2008), and *Ameyamaea* (Yukphan et al., 2009). **Figure 3** shows the phylogenetic relationship among the most common Alphaproteobacterial taxa in our samples, demonstrating a high diversity of *Acetobacteraceae*. However, the high predominance of Alphaproteobacteria and *Acetobacteraceae* at the class and family levels was driven by the consistent occurrence and high relative abundance of a few single phylotypes (**Figures 4A,B**). One single phylotype (1045), which is 97% similar to *G. diazotrophicus*, was the most common in all our samples. The exact phylogenetic placement of phylotype 1045 cannot be completely resolved with the 16S V-5/V-6 region used in this study (**Figure 3**), and may represent a novel species in the genus *Gluconacetobacter* or a related genus within the *Acetobacteraceae*.

The relative abundance and identity of the rest of the dominant endophytic taxa differed somewhat between the two conifer species, although there was also substantial overlap. The most prominent difference was seen between WE and treeline individuals, rather than between the two conifer species, although our small sample size of treeline trees (two trees of each species) prohibits us from drawing any conclusions based on these. Overall, the community of dominant taxa in *P. flexilis* was less diverse; in addition to 1045 only one more phylotype (1516) was consistently present above 10% in the WE samples, and another (1068) consistently present above 4% (**Figure 4A**). Both phylotypes 1516 and 1068 belong to the *Acetobacteraceae*. Phylotype 1516 is 95% similar to *Gluconacetobacter liquefaciens*, and phylotype 1068 is 98% similar to *Tanticharoenia sakaeratensis.* The rest of the 10 most abundant phylotypes in *P. flexilis* WE samples were present at much lower relative abundance (**Figure 4A**). An analysis of all phylotypes in all samples ensured that no individual sample was dominated by a phylotype other than the 10 most abundant in each tree species (data not shown).

The two conifer species shared five of their 10 most dominant phytotypes (**Figures 4A,B**). *P. engelmannii* WE samples had a slightly more diverse community of dominant phylotypes, with more phylotypes present above 4%, and with more variation in dominant phylotypes within and among individuals. Like *P. flexilis*, *P. engelmannii* had a high relative abundance of phylotype 1516 (5–16%). *P. engelmannii* trees at WE sites were also dominated by phylotypes not found in abundance in treeline *P. engelmannii* or *P. flexilis* samples; phylotype 1507 is a *Methylobacterium* species; 1295 is 95% similar to *Terriglobus roseus* in the family *Acidobacteria*; and 1324 is 95% similar to *Kozakia balinesis*, another species in the family *Acetobacteraceae* (**Figure 4B**).

Phylotypes shared across all individuals in one conifer species but not found in the other conifer species were rare (**Figure 5**). Only a limited subset of the needle endophytic microbiota appeared to be host species-specific; instead the differences in endophytic community composition between the two hosts were due to different relative abundances of a set of shared dominant phylotypes. The relative abundance of phylotype 1045 was highest in trees growing at treeline, making up 53 and 49% of the sequences in the *P. flexilis* treeline individuals, and 38 and 39% of the sequences in the *P. engelmannii* treeline individuals (**Figures 4A,B**). In the two *P. flexilis* treeline individuals, phylotype 1644, which is 97% similar to *Asaia lannaensis* (also in the *Acetobacteraceae* family), was the second most common after phylotype 1045. One *P. engelmannii* individual at treeline also had an increased relative abundance of phylotype 1644. The other *P. engelmannii* treeline individual had an increased relative abundance of a different phylotype—816, which is 99% similar to *Pandoraea pnomenusa* in the *Burkolderiales* family.

### STRUCTURE OF CONIFER NEEDLE ENDOPHYTE COMMUNITIES

Principal coordinate analysis of the unweighted UniFraq diversity distance matrix demonstrated that within the WE habitat, host species identity controls endophyte community composition (**Figure 6**). *P. flexilis* samples from the two WE locations clustered to the exclusion of *P. engelmannii* samples from the same locations. Conifer needle endophyte community structure may also be a function of elevation, as the endophyte communities from our treeline samples clustered together to the exclusion of communities from WE samples. However, endophytic communities from more treeline samples will need to be characterized to confirm this observation.

## DISCUSSION

Our results demonstrate that *P. flexilis* and *P. engelmannii* growing at Niwot Ridge are consistently colonized by a limited set of bacterial species belonging to the Alphaproteobacteria and the Acidobacteria, and that the two species share some of the most dominant phylotypes. Most remarkably, all needle samples of both conifer species were dominated by the same phylotype, a species most closely related to the N_2_ fixing species *G. diazotrophicus*. However, is important to point out that 97% similarity over the V-5/V-6 region is not sufficient to identify a particular species. The primer pair used in this study have been used with other plants without resulting in a similar dominance across samples of one phylotype (Bodenhausen et al., 2013, Carrell and Frank, unpublished). Therefore, the patterns observed here are not likely to be caused by primer bias. It is also possible that specific taxa could be selected by our treatment (e.g., transportation in plastic bags at 4°C). However, this is unlikely since endophyte communities of other conifer tissues and species treated the same way are not consistently dominated by a few taxa (Carrell and Frank, unpublished). In addition, we find the same pattern in needles of *P. contorta* where the time from sampling to sterilization was less then 2 h (Carrell and Frank, unpublished).

Bacterial species that are consistently recovered from a certain host species are predicted to be critical to the function of the microbial community (Shade and Handelsman, 2012). For host-associated communities, this may translate into distinct functional roles for those species within the host. However, abiotic factors could also shape host-associated bacterial communities, contributing to consistency in microbiota across samples if the environment is consistent. The dominance of Acidobacteria and Alphaproteobacteria in all our samples probably reflects the ability of these bacteria to survive the conditions within conifer needles. In addition, functionally relevant conifer-bacteria partnerships could underlie their dominance in the endophytic community.

The relative abundance of specific phylotypes from the Acidobacteria was low and their presence was not consistent across samples. Given that Acidobacteria have been found to dominate the alpine dry meadow site soils at Niwot Ridge (Lipson and Schmidt, 2004), their overall prevalence might reflect their ability to tolerate the needle environment and their abundance in the soil rather than a functional relationship with the conifer host. Alphaproteobacteria are also common in the soil at Niwot Ridge (Lipson and Schmidt, 2004); however, in contrast to the Acidobacteria, the dominance of Alphaproteobacteria in all our samples was driven by a few specific phylotypes from the *Acetobacteraceae* (e.g., 1045, 1516, and 1644 in *P. flexilis*, and 1045, 1516, 1324, and 1644 in *P. engelmannii*), suggesting that in addition to tolerating the needle environment, those bacterial taxa serve distinct functional roles within the needles of our subalpine conifers. An association based merely on the ability to survive the environment inside pine needles would more likely result in a variable (within and among individuals) community of taxa in the *Acetobacteraceae.*

Several high-throughput surveys of endophytic 16SrRNA have been published recently, but no study to date has reported a similarly simple and consistent endophyte microbiota. A study of the *Populus deltiodes* root endophyte community reported that a *Pseudomonas* phylotype accounted for 34% of bacterial sequences in all samples combined; however, the extent to which this phylotype was consistently associated with individual trees was not reported (Gottel et al., 2011). Similarly, a study of the leaf endo- and epiphytes of *Arabidopsis thaliana* found a high relative abundance of a single *Pseudomonas* phylotype at 10.9% on average in the endophyte community (Bodenhausen et al., 2013), but inter-individual variability in the relative abundance of phylotypes was not studied. Finally, two recent large-scale studies of *A. thaliana* root endophytes did not report consistent dominance of one or a few phylotypes (Bulgarelli et al., 2012; Lundberg et al., 2012), and comparison across *A. thaliana* root microbiomes did not reveal a common core at the phylotype level (Schlaeppi et al., 2014).

It is important to point out that the phylotypes with the highest number of sequences are not necessarily the most abundant in the community. One potential source of bias is the primer used in this study (799f), which was designed to exclude chloroplast sequences, but likely excludes other sequences as well (e.g., the Cyanobacteria). However, because this primer has been used with very different results in terms of community diversity, variability and taxonomic distribution (Bodenhausen et al., 2013; Schlaeppi et al., 2014), it unlikely underlies the community consistency observed here.

The high relative abundance of a few phylotypes across individuals, host species and elevations may be due to ecologically significant associations between conifers and specific bacteria in the family *Acetobacteraceae*. Species in this family are often found as endophytes, with documented functions in N fixation (Boddey et al., 2003), phytohormone production (Lee et al., 2004), and pathogen antagonism (Blanco et al., 2005). *Acetobacteraceae* are also common symbionts of insects (Crotti et al., 2010). A number of strains from the family are known to solubilize phosphate (P; Loganathan and Nair, 2004). Although P solubilizing endophytes can increase plant uptake of P (Verma et al., 2001; Wakelin et al., 2004), this is not a likely function of above-ground endophytes. Therefore, N_2_ fixation, pathogen protection, or phytohormone production is more likely to underlie the high relative abundance of *Acetobacteraceae* inside *P. flexilis* and *P. engelmannii* needles. *G. diazotrophicus*, *Gluconacetobacter johannae*, *Gluconacetobacter azotocaptans*, *Swaminathania salitolerans*, and *Acetobacter peroxydans* all fix N_2_ in association with plants (Gillis et al., 1989; Fuentes-Ramirez et al., 2001; Loganathan and Nair, 2004; Muthukumarasamy et al., 2005), and *Gluconacetobacter kombuchae* and *Acetobacter nitrogenifigens*, are free-living N_2_ fixers (Dutta and Gachhui, 2006, 2007). Among N_2_ fixing endophytes in the *Acetobacteraceae*, *G. diazotrophicus* is probably the best studied. This species colonizes the intercellular spaces of sugarcane stems (Dong et al., 1994), and may, together with other diazotrophic endophytes of sugarcane, provide the host plant with substantial amounts of N (Boddey, 1995). *G. diazotrophicus* has also been found to dominate N_2_ fixation “hotspots” in soil (Reed et al., 2010).

Fertilization experiments in the subalpine forest in Colorado have suggested that this habitat is still N limited, despite increasing N deposition (Rueth and Baron, 2002), raising the possibility that this partnership involves endophytic N_2_ fixation. More research is needed to evaluate the role of phylotype 1045 in our conifer species; however, evaluation of biological N_2_ fixation associated with non-legumes can be challenging (Boddey, 1995). Phylotype 1045 may not be a culturable strain as our initial attempts to culture any *Acetobacteraceae* from needles have been unsuccessful (Wilson and Frank, unpublished). N_2_-fixing potential and expression of nitrogenase in, e.g., soil and ocean water is commonly estimated via amplification of *nifH* with degenerate primers (Farnelid et al., 2013; Collavino et al., 2014). Unfortunately, although a few studies report amplification of *nifH* (nitrogenase) from DNA extracted from plants (e.g., sugarcane, sweet potato and rice roots; Ueda et al., 1995; Reiter et al., 2003; Ando et al., 2005), this has proven challenging in our system.

The idea that conifers form associations with N_2_-fixing bacteria is not new. Early attempts to estimate rates of N_2_ fixation in temperate and boreal forests detected significant amounts of atmospherically derived N in the conifer canopy (Richards, 1964; Jones et al., 1974; Favilli and Messini, 1990). Moreover, natural abundance studies that exploit naturally occurring differences in ^15^N composition between plant-available N sources in the soil and that of atmospheric N_2_, have shown that the *Pinaceae*, which presumably do not fix appreciable amounts of N_2_, run counter to the expectation of high ^15^N abundance and low foliar content for non-N_2_ fixing plants (Delwiche et al., 1979; Virginia and Delwiche, 1982; Näsholm et al., 2009).

Endophytic N_2_ fixation could explain why some gymnosperms are able to grow in extremely N limited environments (Anand et al., 2013). It is generally presumed that conifers and other non-nodulated plants get their N from the soil. Conifer uptake of amino acids and proteins has been demonstrated but its quantitative importance is still under debate (Näsholm et al., 2009). Rhizospheric N_2_ fixation has been suggested as a conifer N source, but the activity has been found too low to support N requirement, at least in pines (Chanway and Holl, 1991). Ectomychorrhizae are another suggested N source (Hobbie et al., 2000; Chapman and Paul, 2012), but work on non-mycorrhizal conifer seedlings provides strong support for endophytic N_2_ fixation as an alternative explanation: Conifer seedlings inoculated with N_2_ fixing *Paenibacillus* strain isolated from *P. contorta* growing in N limited soil (Bal et al., 2012) have been found to acquire significant amounts of seedling foliar N from the atmosphere compared to control seedlings (Bal and Chanway, 2012; Anand and Chanway, 2013).

In light of this, it is striking that several potentially N_2_ fixing taxa dominate the community of needle-associated endophytes of conifers growing in the N limited subalpine environment at Niwot Ridge. Phylotype 1045, which is 97% similar to *G. diazotrophicus*, and the most common in every single sample, is the main candidate for an N_2_ fixing symbiosis in *P. flexilis* and *P. engelmannii* at this location. Interestingly, we found that phylotype 1045 was present in higher relative abundance in *P. flexilis*, which is found on drier and likely lower N soils than *P. engelmannii*, which is found on more mesic and potentially higher N soils. We also found that in both conifer species, the relative abundance of this phylotype was higher in trees growing at treeline than in trees growing at the WE, which could reflect slower N turnover at treeline or higher N demands by treeline trees. However, total abundance of phylotype 1045 may not differ among WE and treeline trees; it may simply be a function of overall lower diversity in treeline environments. More endophyte community data from the treeline environment will be required to establish links between the conifer endophyte community structure and elevation.

A consistent core microbiota similar to the one observed here would be promoted by selective uptake/transfer of bacteria. Not much is known with regards to the transmission of bacterial endophytes in conifer trees, but possible routes include soil, litter, seed and stomata. Given the long lifetime of *P. flexilis* and *P. engelmannii*, as well as needle longevity (e.g., 4–10 years for *P. flexilis*; Schoettle and Rochelle, 2000), the time spent within individual hosts is expected to be longer than for most host–microbe associations, possibly leading to convergence in the endophyte community assembly among different individuals. The consistency in the endophytic community between the two host species is intriguing. This pattern could reflect a strong local and environmental influence on endophyte uptake, leading to a shared endophyte community between co-occurring species in a shared habitat. Alternatively, the selective uptake/transfer of specific strains of *Acetobacteraceae* could be an ancestral trait that is shared between *Pinus* and *Picea* species, which together with *Cathaya* form a clade in the Pinaceae phylogeny (Lin et al., 2010). If so, the dominance of phylotype 1045 in both host species may be the result of a long-standing association with possible co-diversification of host and endophyte. Our current sequences—approximately 300 nucleotides across over the V-5/V-6 region—do not provide enough resolution to evaluate possible divergence between strains represented by phylotype 1045 from the two conifer species. To understand why *P. flexilis* and *P. engelmannii* at Niwot Ridge have such similar endophyte communities, further research is needed that includes a broader range of conifer species, habitats, and geographic locations, along with the amplification of longer segments of the 16S rRNA gene.

This first comprehensive analysis of the bacterial endophyte communities in a gymnosperm provides circumstantial evidence for endophytic N_2_ fixation in subalpine conifers growing in an N limited environment. Demonstration of a role of conifer endophytes in host N acquisition, along with the extent and potential significance in the conifer forest N budget will require direct tests for endophytic N_2_ fixation and N transfer to the conifer host.

## AUTHOR CONTRIBUTIONS

Alyssa A. Carrell and Anna C. Frank conceived and designed the sampling and experiments. Alyssa A. Carrell designed the primers, performed the DNA extraction, PCR amplification, and the data analysis. Anna C. Frank contributed intellectually to data analysis. Alyssa A. Carrell drafted the work; Anna C. Frank revised it critically for important intellectual content.

## Conflict of Interest Statement

The authors declare that the research was conducted in the absence of any commercial or financial relationships that could be construed as a potential conflict of interest.
